# HLA-DQB1*06:02 and a critical amino acid variant in protection against occult hepatitis B virus infection

**DOI:** 10.3389/fimmu.2026.1898993

**Published:** 2026-08-18

**Authors:** Mimi Wu, Wenwen Pi, Qi Li, Yizhen He, Guangshu Yu, Hong Zhu, Zhipan Wu, Ji He, Yanmin He, Faming Zhu

**Affiliations:** 1 Blood Center of Zhejiang Province, Institute of Transfusion Medicine, Hangzhou, China; 2 Medical Test Laboratory, Blood Center of Zhejiang Province, Hangzhou, China

**Keywords:** amino acid variation, haplotype, HLA, HLA-DQB1, occult hepatitis B infection

## Abstract

The human leukocyte antigen (HLA) system plays a critical role in determining the outcomes of hepatitis B virus (HBV) infection, yet the genetic mechanisms underlying occult HBV infection (OBI), a form marked by detectable HBV DNA in the absence of hepatitis B surface antigen, remain poorly defined. Here, we performed high-resolution HLA genotyping and amino acid-based association analysis in a Chinese population comprising 239 OBI cases and 545 healthy controls. We identified the HLA class II allele DQB1*06:02 as a protective signal against OBI, with conditional analysis confirming its independent effect. This allele is carried on the conserved haplotype DRB1*15:01~DQA1*02:01~DQB1*06:02, which was significantly underrepresented in OBI cases. Conversely, the DRB1*09:01~DQA1*03:02~DQB1*03:03 haplotype was associated with increased OBI risk. Analysis of the HLA-DQB1 amino acid residues revealed that position 119, located in an α-helix of the peptide-binding groove, drives a substantial portion of the observed association. The phenylalanine variant at this site remained strongly protective after multiple-testing correction and is encoded by DQB1*06:02. Our study thus delineates both allele and amino acid level HLA determinants of OBI susceptibility, highlighting a key structural residue that may influence antigen presentation and immune clearance of HBV. These findings provide a refined genetic framework for understanding host and virus interactions in occult HBV persistence.

## 1 Introduction

Despite more than three decades of clinical use of the Hepatitis B virus (HBV) vaccine, HBV infection remains a major global health burden (1) and is among the leading causes of chronic hepatitis, liver cirrhosis, and hepatocellular carcinoma worldwide (2, 3). Occult HBV infection (OBI) is a special type of infection that is defined by the presence of replicative HBV DNA in liver tissue and a negative result for hepatitis B surface antigen (HBsAg) in the blood by immunoassay (4). The incidence of OBI among blood donors varies across regions and populations, ranging from approximately 1:1000 to 1:7517 in China (4). These donors are typically aged 20–45 years in China, exhibit normal alanine aminotransferase (ALT) levels, and maintain low-level viraemia, with the concentration of HBV DNA ranging from undetectable to 178 IU/mL (5). OBI is prevalent worldwide and is primarily transmitted through blood transfusion or organ transplantation. This can not only lead to lifelong infection in recipients but also pose a risk of acute reactivation under immunosuppressive conditions, which may progress to severe or even fulminant hepatitis (6, 7). Although the clinical definition of OBI relies on nucleic acid testing, its hallmark feature intermittent, low level viraemia suggests that host immune surveillance plays a decisive role in maintaining this occult state. The ability to control HBV replication below the HBsAg positive threshold varies substantially among individuals, implying the existence of host genetic determinants that modulate the efficiency of antiviral immunity (8). Among these, the human leukocyte antigen (HLA) system is a prime candidate, as it governs the presentation of viral peptides to T cells and thus influences the strength and quality of the adaptive immune response. Therefore, understanding the host genetic basis of OBI is essential for explaining why certain individuals can persistently suppress HBV replication, whereas others progress to overt infection.

The HLA system is highly polymorphic, and 45421 alleles have been identified worldwide according to the IPD-IMGT/HLA database (version 3.65). HLAs are categorised into HLA class I (HLA-A, -B, and -C) and class II (HLA-DR, -DQ, and -DP) (9). HLA class I molecules are expressed on all nucleated cells and present intracellularly derived antigens to cytotoxic (CD8^+^) T cells, leading to the elimination of tumour or infected cells (10). The products of HLA class II loci are expressed on the surface of professional antigen-presenting cells and typically present extracellular peptides to helper (CD4^+^) T cells (11). HLA plays a critical role in regulating immune responses against exogenous infections. This characteristic makes HLA a key candidate biomarker for infectious diseases. A potential association exists between HLA allele polymorphisms and HBV infection (12). Previous studies have shown that HLA-DQB1*03:01 is associated with an increased risk of HBV-related hepatocellular carcinoma in a Chinese Han population (13). In the Shaanxi Han population, the HLA-DRB1*07:01 allele is significantly associated with OBI (14). Furthermore, the haplotype HLA-DRB1*07:01-DQB1*02:02:02 has been shown to confer susceptibility to OBI in the Xi’an Han population, China (15). These findings suggest that specific HLA allele inheritance is a key factor influencing HBV infection outcomes, with HLA-DQB1 and HLA-DRB1 alleles potentially playing important roles in OBI susceptibility or protection. However, the distribution of HLA loci demonstrates significant population-specific and geographic variation, and existing genetic analyses have yielded inconsistent results across different studies. Moreover, recent research on HLA associations with other diseases has indicated that evaluating only classical HLA alleles or single nucleotide variant (SNV) may fail to detect disease risk effects driven by specific amino acid positions, as each amino acid residue is often shared across multiple classical alleles (16, 17). Therefore, amino acid positions may serve as the primary drivers of disease risk or protective effects.

However, current studies investigating the association between HLA and OBI have not reported a potential association between HLA amino acid polymorphisms and OBI susceptibility. In this study, we analysed the HLA class I and II gene profiles of 239 OBI cases and 545 healthy controls to systematically explore associations at the levels of alleles, haplotypes, amino acid positions, and specific residues. These findings are expected to further advance the understanding of the relationship between HLA genetics and OBI.

## 2 Materials and methods

### 2.1 Specimen

Whole blood specimens anticoagulated with EthyleneDiamineTetraacetic Acid (EDTA) were collected from blood donors at the Blood Center of Zhejiang Province, China. All donors were enrolled and tested according to the guidelines for blood donation in China, as described in our previous report (18). The specimens were tested for HBsAg, anti-HCV, anti-HIV, and anti-Treponema pallidum (anti-TP) using enzyme-linked immunosorbent assays (ELISAs) and for HBV DNA, HCV RNA, and HIV RNA using nucleic acid amplification testing (NAT) assays, as described in our previous report (19). HBsAg (–)/HBV DNA (+) specimens were continuously collected from August 2021 to October 2022. The control group was composed of voluntary blood donors whose specimens tested negative for HBsAg, anti-HCV, anti-HIV, and anti-TP; for HBV DNA; and for HCV RNA and HIV RNA. In addition, OBI cases were subjected to quantitative serological testing for HBsAg, HBeAg, anti-HBs, anti-HBc and anti-HBe using chemiluminescence immunoassays (CLIA), following the manufacturer’s instructions. All enrolled donors provided written informed consent for study participation. This study was approved by the Ethics Committee of the Blood Center of Zhejiang Province (Approval No. 2024-004).

### 2.2 HLA genotyping using next-generation sequencing

Genomic DNA was extracted from whole blood specimens using a commercial reagent kit and then genotyped for 11 HLA loci (HLA-A, -B, -C, -DPA1, -DPB1, -DQA1, -DQB1, -DRB1, -DRB3, -DRB4, and -DRB5) using an AllType™ next-generation sequencing (NGS) kit (One Lambda Inc., Canoga Park, CA, USA), as described in our previous report (18). In brief, target sequences at these loci were first amplified using mixed primers, after which library preparation was performed using the Ion Shear Plus Reagents Kit and Ion Plus Fragment Library Kit (One Lambda, Inc.). The final libraries were subsequently sequenced on an Ion Torrent S5 platform (Thermo Fisher Scientific, Waltham, MA, USA). The genotypes were assigned using TypeStream Visual software version 2.0 (One Lambda, Inc.) according to the manufacturer’s instructions.

### 2.3 Determination of the amino acid sequence of DQβ1 and modeller

On the basis of the HLA-DQB1 alleles, the amino acid sequences of the DQβ1 chains were extracted from the IMGT/HLA public database (http://www.ebi.ac.uk/ipd/imgt/hla/). A structure model of the HLA-DQB1*06:02 molecules was constructed based on the 3D structure of Protein Data Bank (PDB) ID: 6DIG. Structure alignment analysis and the labelling of active and interaction site residues were performed using PyMOLWin EdU according to the manufacturer’s instructions.

### 2.4 Statistical analysis

All analyses between haplotypes, alleles, and amino acids in HLA class II genes, including calculations of the Hardy-Weinberg Equilibrium (HWE), confidence intervals (CIs), odds ratios (ORs), and P values, were performed in R version 4.4.3. Associations of HLA alleles with OBI were tested using logistic regression. Conditional analyses were performed by adding specific alleles or variants of interest as covariates. The frequencies of HLA alleles associated with specific amino acids were computed using the BIGDAWG R package. Haplotype estimations and association analyses with the disease were performed using the haplo.stats R package. Principal Component Analysis (PCA) was performed based on Single Nucleotide Polymorphism (SNP) data to evaluate genetic relatedness among samples and to detect potential population stratification. The distribution of samples in the principal component space provided a visual representation of genetic distances, enabling the identification of samples with close genetic relationships, potentially indicative of cryptic relatedness as well as outliers with divergent genetic backgrounds. These results informed subsequent quality control procedures and were used to adjust for confounding due to population stratification in association analyses. To ensure statistical robustness and to avoid unstable effect estimates due to sparse data, the primary haplotype regression analyses were restricted to haplotypes with a frequency of ≥ 1% in either the OBI group or the control group. Correction for multiple testing was performed using the Bonferroni method.

## 3 Results

### 3.1 Fifteen HLA alleles were associated with OBI

**Figure 1:**
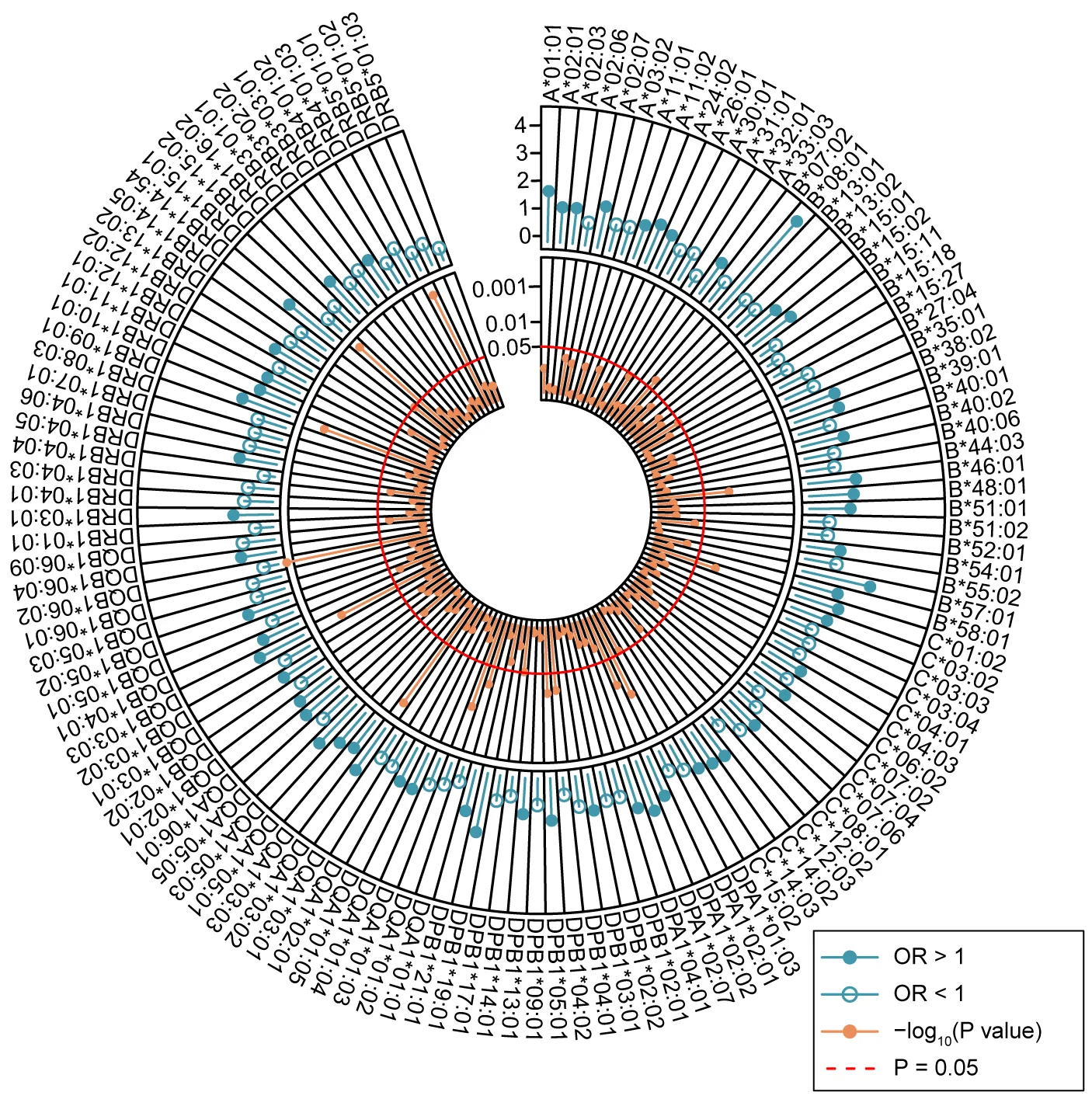
Association analysis results for all HLA class I and class II alleles. The results from the association analysis of all identified HLA class I and II alleles are presented in this circular figure. The alleles are positioned around the circumference. A statistically significant P value is encoded by the radial distance from the plot centre, where increased distance corresponds to a lower P value. The red concentric line indicates the P = 0.05 cutoff. The effect size expressed as the odds ratio (OR) is represented by points, with solid points for OR>1 and open points for OR<1.

A total of 239 blood specimens from OBI carriers and 545 healthy controls were analysed. The distribution of the genotypes was fitted by the HWE test in the participants with OBI and controls. A total of 270 HLA alleles at the second-field resolution were successfully obtained using NGS, including 32 HLA-A, 75 HLA-B, 34 HLA-C, 43 HLA-DRB1, 18 HLA-DQA1, 18 HLA-DQB1, 7 HLA-DPA1, 29 HLA-DPB1, 4 HLA-DRB3, 4 HLA-DRB4, and 6 HLA-DRB5 alleles. Fifteen HLA alleles were significantly associated with OBI (P<0.05; **Figure 1**; **Table 1**). However, only nine HLA class II alleles were still significantly associated with OBI after the Bonferroni correction (P<0.05; **Table 1**), comprising five protective and four susceptible alleles. The most significantly associated alleles included the protective alleles HLA-DQB1*06:02 and HLA-DRB5*01:01, as well as the susceptible alleles HLA-DQA1*03:02 and HLA-DQB1*03:03 after the Bonferroni correction (**Table 1**).

**Table 1:** Association results for HLA allele analysis in Chinese people with OBI (P<0.05).

Allele	Allele Frequency in OBIs (n=239)	Allele Frequency in Controls (n=545)	OR	P value	Pc value*
Protective alleles					
DQB1*06:02	0.031381	0.088991	0.33	0.000097	0.001742
DRB1*15:01	0.054393	0.114679	0.44	0.000272	0.011682
DRB5*01:01	0.075314	0.140367	0.50	0.000337	0.001180
DPA1*02:01	0.081590	0.133945	0.57	0.004060	0.023799
DQA1*01:02	0.108787	0.167890	0.60	0.003267	0.024648
DQA1*01:01	0.012552	0.035780	0.34	0.015354	0.092126
DPB1*04:02	0.018828	0.044037	0.41	0.016110	0.233596
Susceptible alleles					
DQA1*03:02	0.221757	0.148624	1.63	0.000431	0.007757
DQB1*03:03	0.232218	0.161468	1.57	0.000904	0.008138
DPA1*02:02	0.550209	0.477982	1.34	0.009927	0.029895
DRB1*09:01	0.217573	0.146789	1.62	0.000607	0.013056
DPB1*05:01	0.426778	0.357798	1.32	0.013725	0.233596
C*01:02	0.213389	0.161468	1.41	0.013476	0.458214
B*46:01	0.148536	0.102752	1.52	0.009712	0.728417
B*08:01	0.014644	0.003670	4.04	0.026616	0.853108

*Pc value = Bonferroni-corrected P value.

### 3.2 HLA-DQB1*06:02 drives the protective signal for OBI

**Figure 2:**
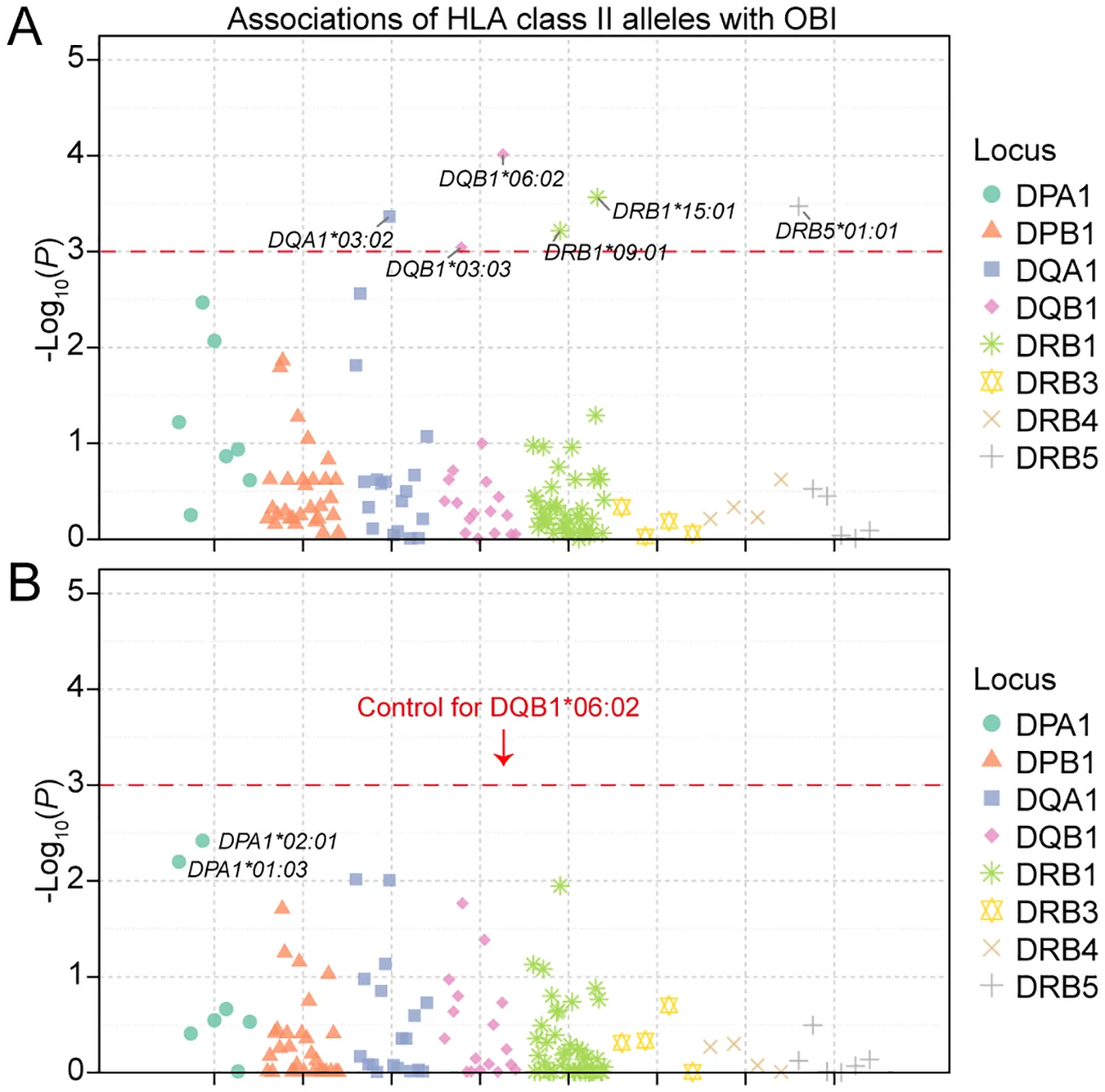
Associations of HLA class II alleles with OBI. **(A)** Association results for HLA class II allele analysis with OBI. Significant associations were observed for HLA-DQB1*06:02, HLA-DRB1*15:01, and HLA-DRB5*01:01 (protective), as well as for HLA-DQA1*03:02 and HLA-DQB1*03:03 (risk). **(B)** After adjusting for HLA-DQB1*06:02, no HLA alleles were significantly associated with OBI. A P value of <0.001 was adopted as the threshold for statistical significance in the classic HLA allele association analysis.

A total of 270 distinct alleles were identified across both OBI patients and healthy controls, and the threshold was set as P<0.001 (P<0.05/270) for unconditional analysis. It was determined that HLA-DQB1*06:02 was significantly associated protective allele with OBI (**Figure 2A**; **Table 1**), which was significantly less frequent in OBI patients than in healthy controls. After conditioning for HLA-DQB1*06:02, no additional HLA class II alleles remained significantly associated with OBI at the predefined threshold of P<0.001 (**Figure 2B**). Notably, neither HLA-DPA1*02:01 nor HLA-DPA1*01:03 showed an independent association after adjustment for HLA-DQB1*06:02. These results reinforce that HLA-DQB1*06:02 is the primary driver of protection in this cohort.

### 3.3 Linkage disequilibrium among HLA alleles

**Figure 3:**
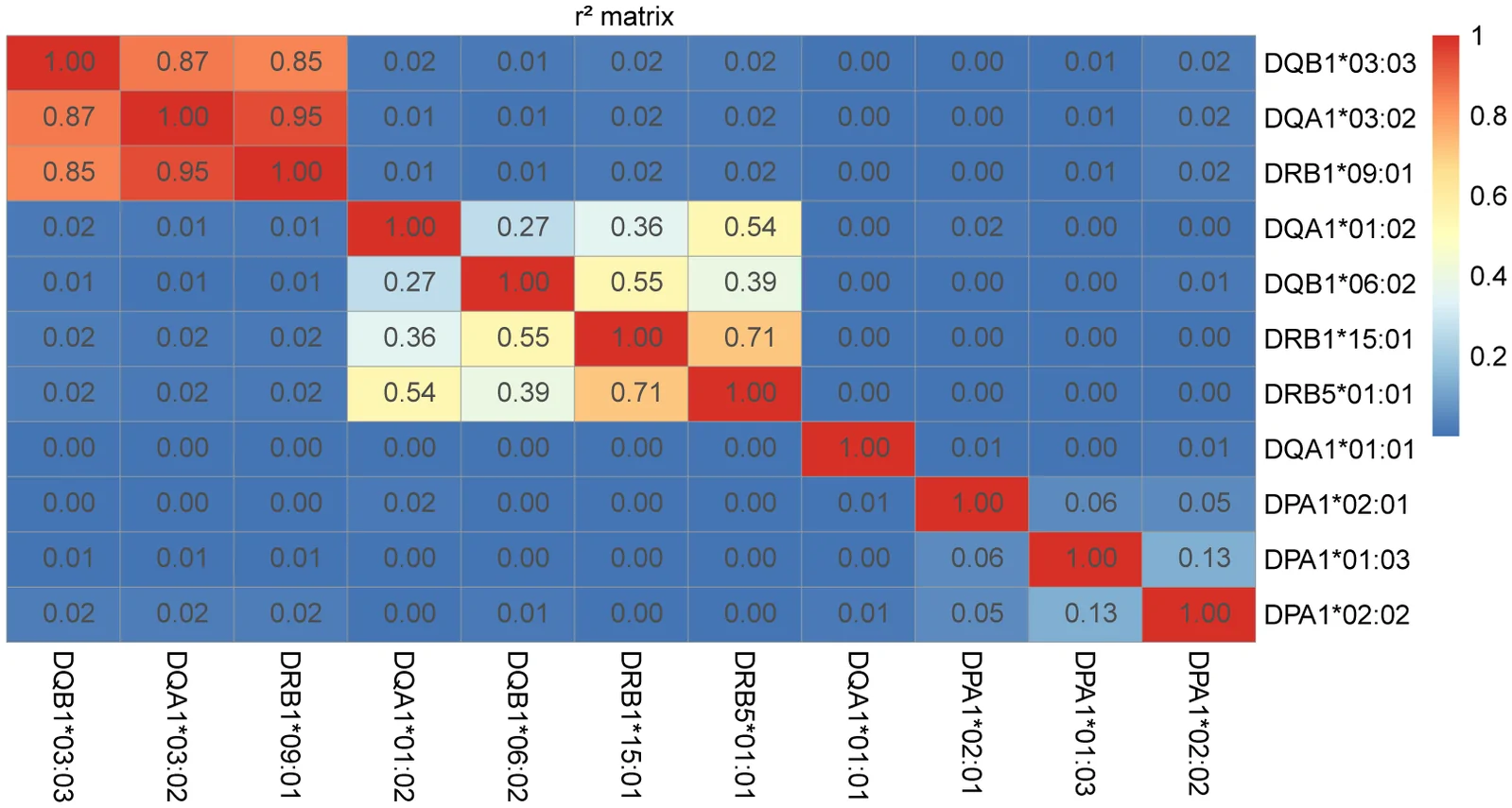
Pairwise linkage disequilibrium (r²) plot of HLA alleles associated with OBI. HLA alleles have been clustered according to their pairwise linkage disequilibrium on both the x- and y-axes. On the left-hand y-axis and the top x-axis are labelled risk or protective alleles in OBI.

The HLA region contains the densest LD in the human genome; therefore, the LD was also analysed in OBI (**Figure 3**; **Table 2**). Strong LD was observed among HLA-DQB1*06:02, HLA-DRB1*15:01, and HLA-DRB5*01:01, with D′ values approaching 1.0 and r² values ranging from 0.39 to 0.71, indicating that these three alleles reside on the same conserved protective haplotype. In contrast, HLA-DPA1*01:03 showed virtually no LD with HLA-DQB1*06:02 (D′=0.0236, r²=0.0000), and both HLA-DPA1*02:01 and HLA-DPA1*01:03 exhibited negative LD with HLA-DQB1*06:02 (D′=-0.5825 and -0.5941, respectively), albeit with low r² values. These patterns suggest that the two HLA-DPA1 alleles are independent of the major protective haplotype and may represent distinct genetic signals.

**Table 2:** LD analysis among the susceptible HLA class II alleles in OBI.

Allele-1	Allele-2	D’	r^2^
DQB1*06:02	DRB1*15:01	1	0.5485
DQB1*06:02	DRB5*01:01	1	0.3901
DRB1*15:01	DRB5*01:01	1	0.7113
DQB1*06:02	DPA1*01:03	0.0236	0.0000
DQB1*06:02	DPA1*02:01	-0.5825	0.0043
DPA1*02:01	DPA1*01:03	-0.5941	0.0557

### 3.4 HLA DR-DQ haplotypes associated with OBI

The primary protective association was unequivocally mapped to the HLA-DQB1 locus, and DR-DQ haplotypes have been consistently implicated in HBV infection outcomes. Thus, we focused the extended haplotype analysis on the HLA-DRB1~DQA1~DQB1 region. Extended haplotype analysis across HLA-DRB1, -DQA1, and -DQB1 revealed a complex landscape of genetic predisposition to OBI. Consistent with the allele association results, the haplotype HLA-DRB1*09:01~DQA1*03:02~DQB1*03:03 was significantly enriched in OBI cases compared with controls (OR = 1.96, 95% CI: 1.26-3.06; P = 0.00376), supporting its role as a risk-conferring haplotype. In contrast, the haplotype HLA-DRB1*15:01~DQA1*02:01~DQB1*06:02 was absent in OBI patients and showed a significantly reduced frequency in cases compared with controls (OR = 0.078, 95% CI: 0.005-1.303; P = 0.00782), underscoring its strong protective effect.

Beyond these predominant signals, several rare haplotypes were observed exclusively or at markedly elevated frequencies among OBI cases. Notably, HLA-DRB1*16:02~DQA1*01:02~DQB1*05:02 was present only in the OBI group, with a frequency of 0.84%. Similarly, HLA-DRB1*14:54~DQA1*05:05~DQB1*05:02 was detected exclusively in OBI patients, with a frequency of 0.63%, and HLA-DRB1*16:02~DQA1*03:02~DQB1*05:02 showed a nine fold increased frequency in OBI cases. Additionally, the HLA-DRB1*12:02~DQA1*06:01~DQB1*03:02 haplotype was more common in OBI patients than in controls (**Table 3**). Taken together, these findings illustrate the diversity of HLA haplotypes associated with OBI susceptibility and protection, which may contribute to the genetic architecture of occult HBV infection.

**Table 3:** Extended haplotype analysis among HLA-DRB1, -DQA1, and -DQB1 in Chinese people with OBI (P<0.05).

Haplotypes	Control frequency	OBI frequency	OR (95% CI)	P value
DRB1*09:01~DQA1*03:02~DQB1*03:03	0.04220	0.07950	1.96 (1.257-3.055)	0.00376
DRB1*15:01~DQA1*02:01~DQB1*06:02	0.01280	0.00000	0.078 (0.005-1.303)	0.00782
DRB1*16:02~DQA1*01:02~DQB1*05:02	0.00000	0.00840	20.684 (1.111-384.959)	0.00856
DRB1*07:01~DQA1*01:02~DQB1*02:02	0.01740	0.00210	0.118 (0.016-0.885)	0.02460
DRB1*14:54~DQA1*05:05~DQB1*05:02	0.00000	0.00630	16.054 (0.828-311.417)	0.02820
DRB1*16:02~DQA1*03:02~DQB1*05:02	0.00090	0.00840	9.19 (1.024-82.443)	0.03240
DRB1*12:02~DQA1*06:01~DQB1*03:02	0.00370	0.01460	4.035 (1.176-13.85)	0.04130

### 3.5 Association of HLA-DQB1 amino acid variants with susceptibility to OBI

**Figure 4:**
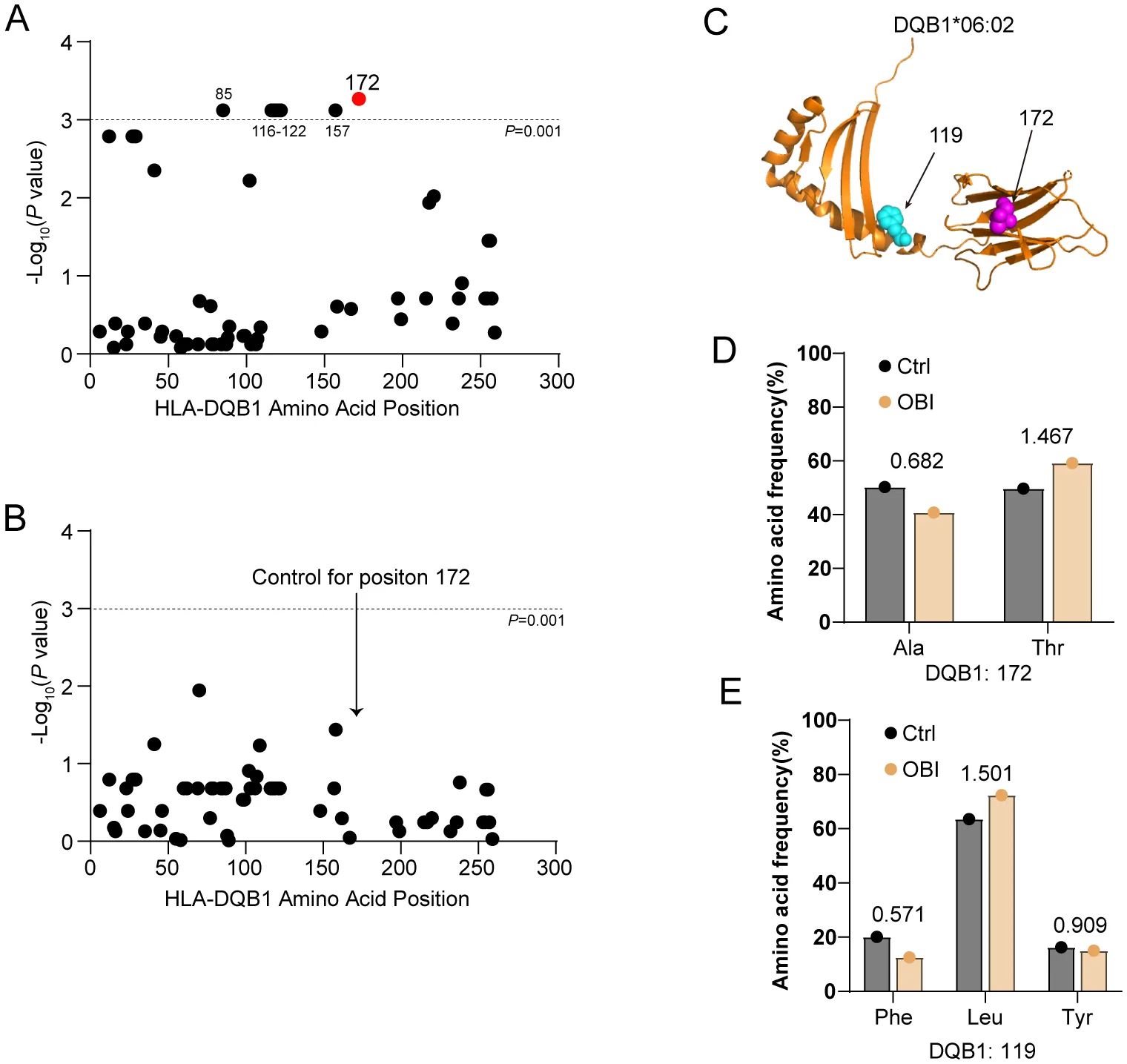
Association of MHC class II molecules with OBI. **(A)** Unconditional association analysis revealed that amino acid positions 172 and 119 in the HLA-DQβ1 chain were most significantly associated with OBI risk. **(B)** After adjusting for position 172, no amino acid position was significantly associated with OBI. **(C)** Location of amino acid positions 119 (blue) and 172 (magenta) on the surface of the HLA-DQ peptide-binding groove (PDB ID: 8VDU). **(D)** Effects of individual amino acid residues at position 172 in HLA-DQβ1. The frequencies of alanine (Ala) and threonine (Thr) at this position in OBI patients and controls are plotted, together with the corresponding univariate ORs. **(E)** Effects of individual amino acid residues at position 119 in HLA-DQβ1. The frequencies of phenylalanine (Phe), leucine (Leu), and tyrosine (Tyr) at this position in OBI patients and controls are plotted, together with the corresponding univariate ORs.

To elucidate the precise structural sites mediating the association between HLA-DQB1 and OBI, we analysed the correlation between individual amino acid positions in the HLA-DQB1 molecule. We identified 60 positions in the OBI patients and healthy controls; therefore, we set P<0.05/60 (P<0.001) as the threshold for statistical significance after the Bonferroni correction. Notably, nine positions were significantly associated with OBI in HLA-DQB1: positions 85, 116-119, 121-122, 157, and 172 (**Figure 4**; **Table 4**). A detailed amino acid-level analysis for each position is provided in **Table 4**.

**Table 4:** Amino acid positions significantly associated with OBI.

Position	OR	CI.lower	CI.upper	P value	Pc value
85	0.666	0.522	0.848	0.000755	0.045
116	0.666	0.522	0.848	0.000755	0.045
117	0.666	0.522	0.848	0.000755	0.045
118	0.666	0.522	0.848	0.000755	0.045
119	0.666	0.522	0.848	0.000755	0.045
121	0.666	0.522	0.848	0.000755	0.045
122	0.666	0.522	0.848	0.000755	0.045
157	0.666	0.522	0.848	0.000755	0.045
172	1.467	1.174	1.836	0.000541	0.032

*Pc value = Bonferroni-corrected P value.

The most significant association after correction was observed at position 172, followed closely by position 119 (**Figure 4A**; **Table 4**). The amino acid residue at position 172 is involved in the formation of a β-sheet in the MHC-DQβ1 chain. Among the two possible amino acid residues, Ala172 has a protective effect, and Thr172 has a risk effect on OBI (**Table 5**). However, after Bonferroni correction, neither association reached statistical significance, suggesting that position 172 may be a potential risk site whose functional amino acid variant requires further confirmation, indicating that the position-level signal at this site does not arise from a single dominant residue (**Table 5**). In contrast, at position 119, Phe119 residue remained strongly protective after correction, whereas Leu119 and Tyr119 showed no significant association. This distinction highlights that position 119, rather than position 172, contains a single functional residue that drives the protective effect, whereas the signal at position 172 may reflect a composite of multiple weaker effects or linkage disequilibrium with position 119 (**Figure 4B**; **Tables 4**, **5**).

**Table 5:** Effects of individual amino acid residues at positions 119 and 172 on the HLA-DQB1 chain.

Position and Amino Acid	Ctrl Frequency(%)	OBI Frequency(%)	Observed Classical HLA-DQB1 Alleles	OR	P value	Pc value
119						
Phe	20.09	12.55	06:01, 06:02, 06:03,	0.571	0.000321	0.043335
Leu	63.58	72.38	02:01, 02:02, 03:01, 03:02, 03:03, 03:05, 03:13, 04:01, 04:02,	1.501	0.000755	0.101925
Tyr	16.33	15.06	05:01, 05:02, 05:03, 05:10, 06:04, 06:09	0.909	0.549651	>0.99
172						
Ala	50.28	40.79	02:01, 02:02, 05:01, 05:02, 05:03, 05:10, 06:01, 06:02, 06:03, 06:04, 06:09	0.682	0.000541	0.073035
Thr	49.72	59.21	03:01, 03:02, 03:03, 03:05, 03:13, 04:01, 04:02,	1.467	0.000541	0.073035

*Pc value = Bonferroni-corrected P value.

The two protective amino acid residues, Phe119 and Ala172, are encoded by the HLA-DQB1*06:02 allele; 12.55% of the OBI patients carried the Phe119 residues compared with 20.09% of the healthy individuals and 40.79% of the OBI patients carried the Ala172 residues compared with 50.28% of the healthy individuals. These findings are consistent with our initial observation that HLA-DQB1*06:02 mediates protection against OBI (**Figure 2A**). The large number of HLA-DQB1 variants poses a challenge for statistical analysis, limiting the power of fine‑mapping at the allele level and increasing the risk of false positives due to multiple testing. Nevertheless, by focusing on key amino acid positions, aggregating variant information across each site, and integrating the biological context, the evidence strongly supports the association signals initially observed at the high-resolution allele level.

## 4 Discussion

Polymorphic variations within the HLA class I and class II gene clusters are critical components of immune responses and serve as key genetic determinants of the immunological profile that dictates HBV persistence versus clearance. Notably, specific polymorphisms within HLA loci, with a particular emphasis on HLA class II, are associated with spontaneous viral clearance or the acquisition of immunity against HBV infection. In this study, we identified a protective allele, HLA-DQB1*06:02, which carried a haplotype and key amino acid residues Phe119 associated with OBI in a Chinese population may influence antigen presentation and immune clearance of HBV.

The HLA-DQB1*06 allele has been linked to the maintenance of long-term immune responses following infant HBV vaccination (20), as well as to chronicity after HBV infection (21). In diverse populations, it has been associated with protection against HBV infection and the modulation of disease progression (22, 23). Studies on the role of HLA-DQB1 gene polymorphisms in HBV infection have shown elevated mRNA levels of HLA-DQB1*06:02 in HBV-infected groups, which may reflect an upregulation aimed at enhancing recognition and clearance of HBV antigens (23). Research on the correlation between HLA-DQB1 allele polymorphisms and HBV infection in the Chinese Han population revealed that the HLA-DQB1*06:02 allele is significantly associated with an enhanced antibody response to hepatitis B. Specifically, HLA-DQB1*06:02 exhibited high specificity in predicting the antibody response to the hepatitis B vaccine (HepB), although all evaluated alleles showed low sensitivity for this prediction (24).

The HLA-DQB1*06:02 allele has been implicated in various pathological contexts beyond HBV infection. Notably, its presence is associated with several autoimmune and immune-mediated disorders, including narcolepsy, multiple sclerosis, systemic lupus erythematosus, sarcoidosis, and primary sclerosing cholangitis. Conversely, HLA-DQB1*06:02 strongly protects against type 1 diabetes (T1D) (25). The mechanism by which HLA-DQB1*06:02 increases the risk for narcolepsy is thought to involve the presentation of specific yet unidentified antigens, positioning it as a potential genetic marker for predicting such individual susceptibility (26, 27). The HLA-DQB1*06:02 allele is associated with an increased risk of multiple sclerosis (MS) in populations from southern Morocco (28). Recent studies in a Romanian population analysing HLA genes in chronically HBV-infected patients reported that HLA-DQB1*06:02 confers significant protection against HBV infection. That study also identified HLA-DRB1*15:01:01 as another potentially protective allele (29). Our study also revealed HLA-DRB1*15:01 as a potential protective factor against OBI. Additionally, HLA-DRB1*07:01 was significantly associated with OBI, indicating susceptibility. Analysis of individual amino acid contributions to disease risk revealed that residues Gln4β, Val57β, Ser60β, and Val78β were linked to susceptibility, whereas Arg4β, Asp57β, Tyr60β, and Tyr78β showed protective associations (14). Our findings further demonstrate that the HLA-DQB1*06:02 allele is more frequent in healthy controls than in the OBI group. The key advance of our study is the fine-mapping of the protective effect to a single amino acid residue, Phe119, rather than merely reporting a broad allele association. Position 119 resides in the α-helical segment that forms one wall of the HLA-DQβ1 peptide-binding pocket. Substitution of phenylalanine by leucine or tyrosine at this site alters both the side-chain hydrophobicity and spatial geometry, which is predicted to differentially affect the repertoire of HBV-derived peptides that can be stably bound and presented to CD4^+^ T cells. In OBI, where HBV DNA persists at low levels despite HBsAg negativity, the efficiency of CD4^+^ T-cell help is critical for sustaining cytotoxic CD8^+^ responses and antibody production (30). We therefore propose that the Phe119 variant may facilitate preferential presentation of immunodominant HBV epitopes, thereby eliciting robust, polyfunctional T-helper responses that reinforce viral suppression below the HBsAg-positive threshold. Several lines of evidence from prior experimental and structural studies support the functional importance of this residue. For instance, comparative peptide-binding assays and homology modeling between HLA-DQA1*01:02-DQB1*06:02 and the closely related DQB1*06:04 allelic protein have demonstrated that amino acid substitutions in the β-chain α-helix significantly alter peptide-binding motifs and specificities (31, 32). Likewise, fine-mapping studies in other diseases, including T1D and multiple sclerosis, have repeatedly shown that single residue substitutions in the α-helical region of HLA-DQβ1 are sufficient to drive disease association (33). Collectively, these observations provide a coherent structural and functional framework for interpreting our genetic finding.

While nonsynonymous variants that alter a protein’s amino acid sequence are generally neutral in most genes, they play a critical role in HLA genes. Within these genes, variations in the antigen-recognition domain directly shape the repertoire of peptides that the HLA protein can present to the T-cell receptor. In this study, we examined the importance of HLA heterozygosity and its protective role against HBV infection. In summary, we identified the independent protective allele HLA-DQB1*06:02 and its haplotype HLA-DRB1*15:01~DQA1*02:01~DQB1*06:02. The amino acid at position 119 within the peptide-binding groove of HLA-DQβ1 likely influences antigen binding, thereby reducing susceptibility to OBI. However, the modest sample size and lack of an independent replication cohort call for larger, multi-ethnic validation studies. Peptide binding assays, antigen presentation assays, and HLA transgenic mouse models are urgently needed to confirm the causal role of Phe119. Further functional studies are warranted to elucidate the mechanisms by which HLA-DQB1*06:02 exerts its protective effect.

Beyond its biological interpretation, our findings have several potential clinical and translational implications that merit consideration. The identification of HLA-DQB1*06:02 as a protective allele and particularly the fine-mapping to the functional residue Phe119 may eventually contribute to individual risk stratification for OBI (29, 34). However, the predictive utility of a single HLA allele is inherently limited, and its integration with other host genetic, viral, and environmental factors would be required to develop a clinically meaningful risk score. In transfusion medicine, donor HLA typing could, after prospective validation in large-scale transmission cohorts, serve as an ancillary tool to estimate residual OBI transmission risk, particularly in settings where nucleic acid testing capacity is constrained (35). HLA genotyping might similarly aid in identifying OBI carriers at elevated risk of reactivation during immunosuppressive therapy, guiding intensified virological surveillance or prophylactic antiviral intervention (36, 37). Given the established link between DQB1*06:02 and enhanced antibody responses to hepatitis B vaccination, our findings further support the concept that HLA genotyping could inform personalized vaccination strategies (20, 24). More broadly, our molecular dissection of the Phe119 residue provides a structural framework for understanding persistent viral control, which may guide the rational design of peptide therapeutic vaccines aimed at enhancing T-cell immunity in chronic carriers or vaccine non-responders (38). We emphasize that these potential applications remain speculative at present and require independent replication and functional validation before any clinical implementation.

This study has several limitations. The modest sample sizes of both the OBI and control groups limited our ability to detect association signals from rare alleles. Although most healthy donors and OBI cases were recruited from Zhejiang Province, China, potential genetic heterogeneity exists within this population. We were unable to address subtle population stratification through genetic ancestry assessment, which may introduce confounding in analyses even within closely related Han Chinese groups. The lack of an independent replication cohort represents another significant limitation of this work. Currently, no publicly available database contains the requisite combination of high-resolution 11 HLA genotyping and strictly defined OBI phenotype to enable in silico validation, as most public HLA data rely on SNP-based imputation rather than direct NGS-based genotyping. However, we have initiated collaborative efforts with blood centers in other regions to establish an independent replication cohort, and we anticipate completing this validation study in the near future. In addition, a limitation of this study is the absence of anti-HBc data for the healthy control group. While anti-HBc positivity in the Chinese population predominantly reflects past HBV exposure rather than active infection, and all controls were negative for both HBsAg and HBV DNA, comprehensive serological profiling including anti-HBc would have provided additional information regarding prior HBV exposure. However, given the exceptionally high prevalence of anti-HBc in the Chinese general population approximately 49%, excluding anti-HBc positive individuals would have compromised the representativeness of the control group (39). Future studies incorporating complete HBV serological panels are warranted to further explore the interaction between past HBV exposure and HLA-mediated genetic susceptibility to OBI. A critical consideration for the generalizability of our findings is the profound ethnic variation in HLA allele frequencies. The protective allele HLA-DQB1*06:02, identified as the primary signal in our Chinese Han cohort, exhibits marked frequency differences across major populations, being relatively common in East Asians and Europeans but much rarer in Africans and certain South Asian groups. Thus, while our results are likely applicable to other East Asian populations, their direct extrapolation to populations with low frequency of this allele remains uncertain. Nevertheless, our amino-acid-level analysis provides a framework that may partially mitigate this limitation, as cross-ethnic replication studies could test the association of the Phe119 residue with OBI, thereby increasing the feasibility and statistical power of validation in diverse populations.

## Data Availability

The aggregated HLA allele frequency data generated in this study are provided in the supplementary materials of this article. Individual-level genotyping data are not publicly available due to ethical restrictions imposed by the Ethics Committee of the Blood Center of Zhejiang Province and the sensitive nature of human genetic information. De-identified data may be available from the corresponding author upon reasonable request, subject to institutional review and approval.
